# Functional aspects of silent ureteral stones investigated with MAG-3 renal scintigraphy

**DOI:** 10.1186/1471-2490-14-3

**Published:** 2014-01-07

**Authors:** Florian Wimpissinger, Christopher Springer, Amir Kurtaran, Walter Stackl, Christian Türk

**Affiliations:** 1 Department of Urology, Rudolfstiftung Hospital Vienna, Juchgasse 25, 1030 Vienna, Austria; 2 Department of Nuclear Medicine, Rudolfstiftung Hospital Vienna, Juchgasse 25, 1030 Vienna, Austria

**Keywords:** Mercaptoacetyltriglycine, Scintigraphy, Stone disease, Urolithiasis, Ureter, Asymptomatic, Obstruction

## Abstract

**Background:**

To investigate functional aspects of silent ureteral stones with special focus on obstruction and its relationship to renal anatomy. The present study is the first investigation of renal excretory function in patients with silent ureteral stones.

**Methods:**

Patients with primarily asymptomatic ureteral stones underwent a mercapto-acetyltriglycine (MAG-3) renal scintigraphy prior to treatment, in addition to anatomic evaluation of renal units and serum creatinine levels. The primary outcome measure was the presence or absence of obstruction. Secondary outcome measures were kidney anatomy, grade of hydronephrosis, location of stones, stone size, and serum creatinine levels.

**Results:**

During a ten-year period, 14 patients (median age 52.6 years; range 37.3 to 80.7 years) were included in the study. The relative frequency of primarily asymptomatic ureteral stones among all patients treated for ureteral stones in the study period was 0.7%. Eleven renal units showed some degree of hydronephrosis while 3 kidneys were not dilated. On the MAG-3 scan, 7 patients had an obstruction of the ureter, 5 had no obstruction, and 2 had dysfunction of the kidney. A statistically significant correlation was established between the grade of obstruction and stone size (p = 0.02).

**Conclusions:**

At the time of presentation, only 64.3% of the patients revealed an obstruction in the stone-bearing renal unit. The degree of hydronephrosis and renal function were very diverse in this subgroup of patients with ureteral stones. The onset of ureterolithiasis and the chronological sequence of obstruction remain unclear in patients who have never experienced symptoms due to their stones.

## Background

Nephrolithiasis is a common disease with a lifetime incidence of approximately 15%; prevalence and incidence rates are on the increase throughout the world [1-3].

At initial presentation, urinary stones may be associated with symptoms (pain, infection, hematuria), or remain asymptomatic as well as undetectable on radiologic studies of the urinary tract [4-6]. Asymptomatic stones are typically found in the collecting system of the kidney. Primarily asymptomatic (or silent) stones in the ureter have been investigated recently and constitute a very rare sub-category of nephrolithiasis [7,8]. The reasons why ureteral stones remain silent are not clear.

Mercaptoacetyltriglycine (MAG-3) renal scintigraphy is an established method to assess renal function in the obstructed kidney [9]. Its use to determine renal function in ureterolithiasis has been described previously [10]. In the present study we aimed to investigate functional aspects with special focus on obstruction. Patients with silent ureteral stones prospectively received MAG-3 scintigraphy prior to treatment as well as underwent anatomic evaluation of renal units and serum creatinine levels.

## Methods

### Patients

During a nine-year period, patients with asymptomatic (silent) ureteral stones were selected prospectively from all nephrolithiasis patients reporting at a urologic stone center. Inclusion criteria were ureteral stones diagnosed randomly, that had caused no symptoms. Exclusion criteria were symptoms related to ureteral stones, including chronic pain, colic, gross hematuria, or urinary tract infection. The study protocol was approved by the ethics committee of The City of Vienna (Wiener Krankenanstaltenverbund). All patients signed informed consent according to the study protocol.

### Outcome measures

The primary outcome measure was the presence or absence of obstruction on MAG-3 renal scintigraphy in patients with silent ureteral stones. Secondary outcome measures were the morphology of the kidney and the collecting system, location of stones (proximal, mid, distal ureter), stone size, and serum creatinine levels.

### Statistical analysis

Spearman’s rank correlation coefficient was used to assess relationships between the grade of obstruction and the grade of hydronephrosis, serum creatinine levels, stone size, and stone location [11].

## Results

Between 2004 and 2013, 14 patients with silent ureteral stones were included in the study. Among 2012 patients treated for ureterolithiasis during the study period, the frequency of silent ureteral stones was 0.7%. The median age of the 14 patients was 52.6 years (range, 37.3 to 80.7 years). Characteristics of patients and stones are summarized in Table 1. The morphology of the renal collecting system, MAG-3 renal scintigraphy, and laboratory parameters are shown in Table 2.

**Table 1:** Patients and stones

Patients	n = 14
Females:males	2:12
**Age (years)**	
Median	52.6 ±13.6
Range	37.3-80.7
**Stone location**	
Proximal ureter	9
Mid ureter	0
Distal ureter	5
Left:right	9:5
**Stone size (mm)**	
Median	10.0 ±6.3
Range	5-25
**Hydronephrosis**	11 (78.6%)
**Renal parenchymal**	
**Reduction**	2 (14.3%)

**Table 2:** Renal morphology and function/obstruction

				**Renal morphology**			**MAG-3 scan**		
			**Stone size**	**Hydronephrosis**	**Parenchyma**	**sCr**	**Function**		
**Patient No.**	**Sex**	**Age**	**(mm)**	**(grade 1–4)**		**(mg/dL)**	**(stone side)**	**Obstruction**	**Comments**
1	M	57,2	17	3	Normal	1,13	45%	Minimal	
2	M	40,9	15	3	Normal	1,00	38%	No	
3	M	43,5	10	2	Normal	1,15	41%	Moderate	
4	M	58,9	8	3	Normal	0,70	45%	Minimal	
5	M	40,2	7	3	Normal	1,10	47%	No	
6	M	80,7	10	no	Normal	1,14	48%	No	
7	M	47,8	25	3	Reduced	1,00	27%	Moderate	
8	M	42,1	20	2	Normal	1,30	21%	n.a.	No excretory function
9	M	68,4	10	no	Normal	1,00	61%	Severe	
10	M	54,4	5	no	Normal	1,11	49%	No	
11	M	80,3	10	2	Normal	3,20	64%	Moderate	
12	M	37,3	15	3	Reduced	1,20	0%	n.a.	No excretory function
13	F	58,5	7	2	Normal	0,7	48%	Minimal	
14	F	50,7	25	2	Normal	0,64	33%	Moderate	

Relationships between outcome parameters assessed with Spearman’s rank correlation coefficient are given in Table 3. A significant correlation was established between stone size and the degree of obstruction (rho = 0.61, p = 0.02). No statistically significant correlations were observed between the degree of obstruction on the MAG-3 scan and the degree of hydronephrosis (p = 0.84), serum creatinine levels (p = 0.18), and stone location (p = 0.79).

**Table 3:** Correlation of grade of obstruction with grade of hydronephrosis, stone size, serum creatinine level, and stone location (Spearman rank correlation coefficient)

	**Grade of obstruction (MAG-3)**
**Grade of hydronephrosis**	
Correlation coefficient	0.06
*P value*	0.84
**Stone size**	
Correlation coefficient	0.61
*P value*	**0.02**
**Serum creatinine level**	
Correlation coefficient	0.38
*P value*	0.18
**Stone location**	
Correlation coefficient	0.08
*P value*	0.79

## Discussion

Silent ureteral stones are an interesting phenomenon and have been reported only in two publications thus far [7,8]. Before the first description of silent ureteral stones established as a primary diagnosis, asymptomatic ureteral calculi had been studied as residual fragments following the treatment of (primarily symptomatic) ureteral stones [12,13].

The aim of the present study was to identify patients with the primary diagnosis of silent ureteral stones, and investigate excretory function and the morphology of the affected renal units in order to determine why the stones caused no symptoms. MAG-3 scans were used to study excretory function and the grade of obstruction prior to stone treatment [9]. Interestingly, the population of 14 patients with silent ureteral stones exhibited different types of obstruction, grades of hydronephrosis, stone sizes, and kidney function. According to these findings, the absence of symptoms related to a ureteral stone does not mean that the respective renal units are not obstructed. Furthermore, the absence of symptoms is not correlated with the grade of hydronephrosis or kidney function.

The high variability of renal obstruction and impairment of function in this subgroup of patients with silent ureteral stones is well correlated with studies concerning primarily symptomatic ureteral stones [14].

In contrast to silent ureteral stones, Kelleher et al. found a clear correlation between stone size (>5 mm), the presence of obstruction, and impairment of renal function in acute calculus obstruction [15]. In another study of acute obstructive urinary calculi, Gandolpho et al. established a significant association between renal impairment and stone size (1.1 to 2.0 cm) in nearly 70% of patients [16].

Sfakianakis et al. found an acute obstruction in 56.5% of patients presenting with renal colic on MAG-3 scans in the emergency setting [10]. Thus, pain is obviously not directly related with obstruction.

Irving et al. studied renal function on the basis of MAG-3 scans in 54 patients with symptomatic ureteral calculi measuring > 4 mm in size; the stones had been treated conservatively [17]. Irving et al. found that 28% of their patients had “silent loss of function” during follow-up, as established on follow-up scans. In this respect, primarily asymptomatic ureteral stones may be regarded as a form of “chronic obstruction.”

Biancani et al. were the first to study physiologic changes caused by chronic obstruction in an animal model in 1976 [18]. Acute obstruction of the ureter led to a rapid increase in intraluminal pressure and dilation of the diameter of the ureter. Subsequently intraluminal pressure declined, whereas deformation of the urinary tract persisted. Primarily silent ureteral stones are found in a very diverse patient population. At the time of presentation, renal units may be obstructed or not; hydronephrosis may be present or not; and renal function may be impaired or not. In patients with just two dysfunctional renal units at the time of presentation, obstruction appears to diminish to some degree in most cases.

Most recently, Marchini et al. were the second group of scientists who studied primarily silent ureteral stones [8]. In a highly selected cohort of 506 patients with ureteral calculi, silent stones were found in 5.3% (27 patients). The study was focused on preservation of kidney function. Patients were investigated with DMSA scans after treatment of the stones. In nine of the 27 patients, DMSA scans were performed before and after treatment. The authors found impaired kidney function in patients with silent ureteral stones; mean postoperative function on the DMSA scan was 31%. DMSA scans and serum creatinine levels revealed no difference in kidney function before and after treatment.

With regard to secondary signs of obstruction on ultrasound, IVU, or CT and actual obstruction on renal scintigraphy, German et al. showed that morphological changes are not directly correlated with the degree of obstruction – even in patients with renal colic [19]. Only 34% of patients with anatomical signs of obstruction had complete obstruction on renal scintigraphy, whereas 24% of patients with renal colic had partial obstruction and no anatomical signs of obstruction on CT. These findings concur with those obtained in the present study: presumably chronic silent ureteral stones are associated with different degrees of hydronephrosis, renal function, and degree of obstruction on MAG-3 scans.

Eisner et al. found that stones located in the proximal ureter were associated with a greater degree of ureteral dilation compared to those located in the distal ureter [20]. In the present study focused of silent ureteral stones, the degree of hydronephrosis was also higher in proximal ureteral stones (88% hydronephrosis in proximal stones versus 60% in distal stones); however, this finding was not statistically significant. The only parameter that correlated with the grade of obstruction was stone size (p = 0.02). This relationship was first reported by Kelleher et al. in symptomatic obstruction [15]. Furthermore, stone size has been established as an important factor in the planning and outcome of treatment [21,22].

In conclusion, silent ureteral stones are clinically relevant. Silent obstruction may lead to irreversible renal function impairment [12].

### Limitations

The major limitation of the present study is the relatively small number of investigated patients. Ureteral stones with no symptoms constitute a rare subgroup of the nephrolithiasis population – even in high-volume stone centers [7]. Larger samples of patients will be needed to obtain statistically significant results. On the other hand, patients with silent ureteral stones might just be an entirely heterogeneous group with different outcomes, depending on the degree and duration of obstruction.

Examination of kidney function and obstruction in patients with silent ureteral stones represents a snapshot of renal units at the time of presentation. As these patients have never experienced subjective symptoms, the chronological history of stone passage or formation – and its impact on excretory function of the kidney over time – cannot be studied at the time of presentation.

## Conclusion

The present study is the first investigation of functional aspects of silent ureteral stones emphasizing on the presence and grade of obstruction seen on MAG-3 renal scintigraphy. In conjunction with two previous studies on this topic – one focusing on patient characteristics and the other examining kidney function after stone treatment – the natural history of silent ureteral stones may be summed up in two distinct characteristics:

1) Asymptomatic onset of obstruction,

2) Chronic persistence of the stone in the ureter with different degrees of obstruction, morphological changes in the ureter and kidney, and deterioration of renal function.

Silent onset of obstruction appears to be the only event that distinguishes these patients from those with acute symptomatic ureteral calculi. Treatment of silent ureteral stones and evaluation of kidney function are mandatory in all of these cases.

## Abbreviations

MAG-3: Mercapto-acetyltriglycine; DMSA: Dimercaptosuccinic adic; IVU: Intravenous urography; CT: Computed tomography.

## Competing interests

All authors of this manuscript declare that they have no competing interest with regard to the data and discussion of the data published in this manuscript.

## Authors’ contributions

F.W. Study design, inclusion of patients, data collection analysis and interpretation of data, manuscript drafting. C.S. inclusion of patients, manuscript revision. A.K. inclusion of patients, diagnostics (MAG-3 scans), manuscript revision. C.T. inclusion of patients, data acqisition, manuscript revision. W.S. inclusion of patients, manuscript revision. All authors read and approved the final manuscript.

## Pre-publication history

The pre-publication history for this paper can be accessed here:

http://www.biomedcentral.com/1471-2490/14/3/prepub
